# Stumped by rapid symptomatic prostatic regrowth: A case report on a STUMP tumour of the prostate resected with HoLEP

**DOI:** 10.1016/j.ijscr.2019.07.058

**Published:** 2019-07-26

**Authors:** Tareq Al Tell, Lorenzo Marconi, Paul Cathcart, Benjamin Challacombe

**Affiliations:** Urology department, Guy’s and St Thomas’ Hospital, London, UK

**Keywords:** STUMP, Prostate, TURP, HoLEP, Benign

## Abstract

**Introduction:**

Stromal Tumour of Undetermined malignant potential (STUMP) is a rare category of the prostate benign tumours, which has a variable clinical presentation, behavior and different approaches to manage it.

**Case presentation:**

A 57 year-old male presented with voiding symptoms of the urinary tract and an increased PSA (14.7 ng/ml). Ultrasound and multiparametric MRI showed an enlarged prostate of 41 ml. Transurethral resection of the prostate (TURP) was performed with 11 g benign tissue removed. The patient presented one year later with the same symptoms; further ultrasound and parametric MRI demonstrated huge recurrence in the transition zone with a new prostate size of 131 ml.

**Discussion:**

Despite no malignancy being identified in the initial TURP histology, the transition zone prostatic tumour behaved aggressively and recurred in just one year. For the first time in literature, the tumour recurred rapidly to three times the initial size and was removed using Holmium Laser enucleation of the prostate (HoLEP), after which no recurrence was seen in 11 months period.

**Conclusion:**

HoLEP can be used to successfully enucleate STUMP tumours but the clinical behavior of the STUMP in prostate is still not understood well because of its rareness.

## Highlights

• Stromal tumour of Undetermined malignant potential (STUMP) of the prostate is a rare tumour arising from the prostate specialized stroma.
• The patient presented with LUTS, MRI showed prostatic growth, and biopsy showed no malignancy.
• The symptoms were treated by TURP and 11 g were removed of the prostate.
• The tumour recurred within less than a year to three times its original size.
• It is the first time in literature, HoLEP was used to remove the origin of the tumour successfully.

## 1 Introduction

Stromal tumour of uncertain malignant potential (STUMP) of the prostate is an extremely rare tumour arising from the specialized stroma of the prostate that shows a wide spectrum of clinical presentations and behavior, ranging from focal benign lesion to a huge aggressive tumour [1]. This Case shows An aggressive, rapidly recurring STUMP of the prostate growing in size from 40 ml to 131 ml in only one year despite interim Transurethral resection of the prostate(TURP) removing 11.85 g of the prostate. The work has been reported in line with the SCARE criteria [2].

## 2 Case presentation

**Fig. 1:**
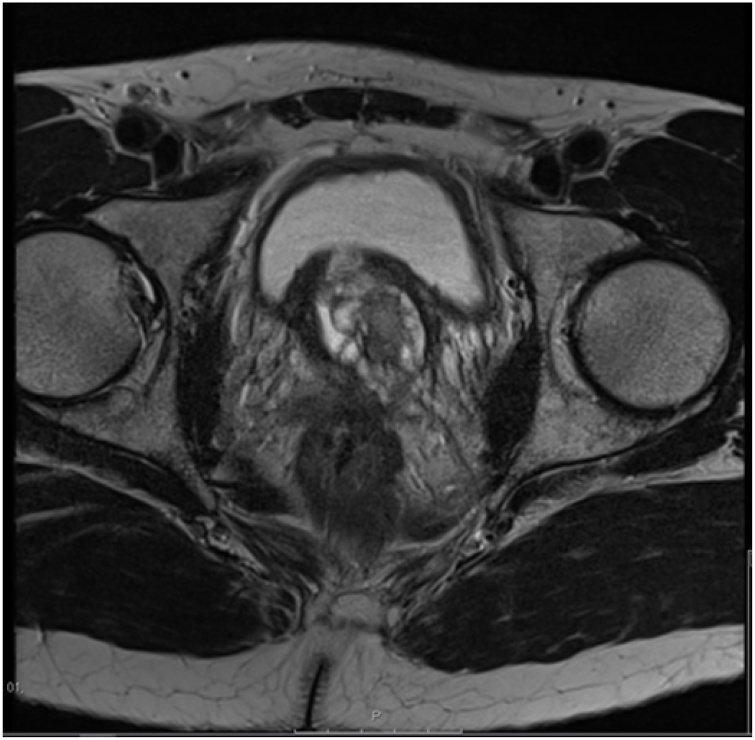
T2-weighted MRI scan (axial view) shows enlarged prostate growing into the bladder base pre-TURP.

**Fig. 2:**
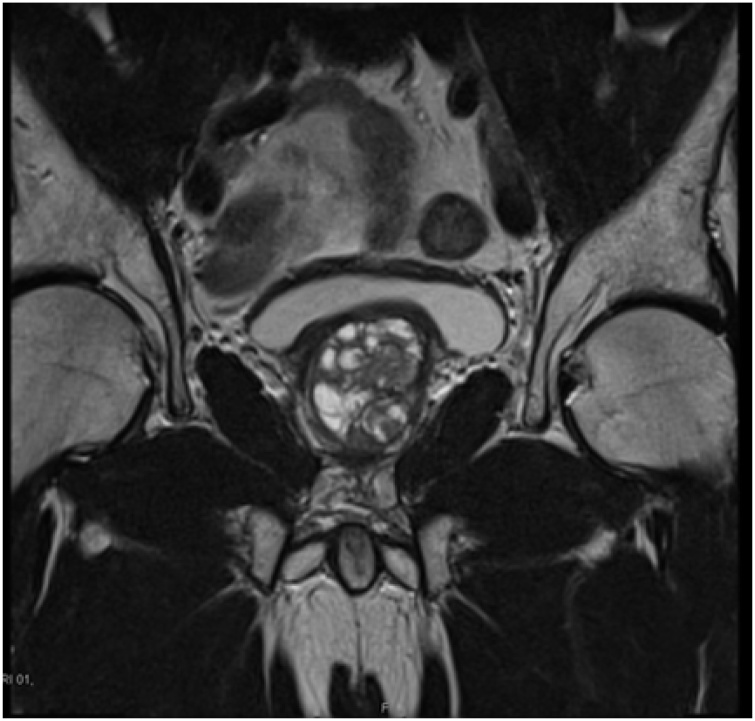
T2-weighted MRI scan (coronal view) shows enlarged prostate growing into the bladder base pre-TURP.

A 57-year-old man with no known medical illnesses, presented with voiding lower urinary tract symptoms (LUTS) of hesitancy, poor flow and incomplete bladder emptying lasting 1.5 months and had a benign feeling prostate on digital rectal examination (DRE). He had previously one episode of urinary retention and had an elevated Prostate Specific Antigen (PSA) of 14.5 ng/ml. He was started on Alfuzosin 10 mg but with no significant improvement in his symptoms. A trans-abdominal ultrasound scan of the urinary tract showed a large prostate gland of size 41 ml, with indentation in the bladder base. A multiparametric MRI scan displayed an enlarged prostate (41 ml) with central gland hypertrophy protruding into the bladder base, the capsule was intact, with normal seminal vesicles, PIRADs 2 score {Fig. 1, Fig. 2}. His repeat PSA was 25 ng/ml.

**Fig. 3:**
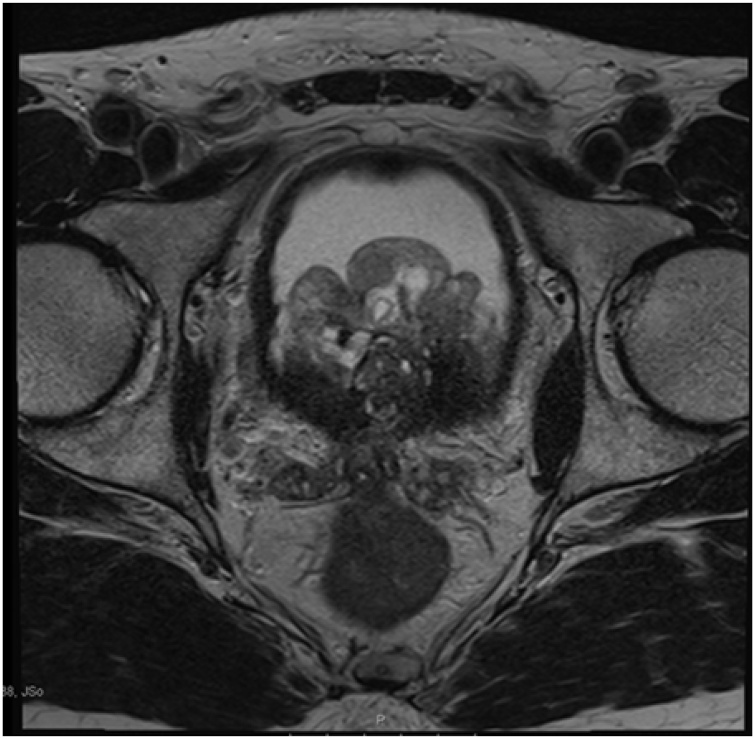
T2-weighted MRI scan (axial view) shows the regrowth of the prostate gland into 131 ml protruding to the bladder base after only one year of TURP.

**Fig. 4:**
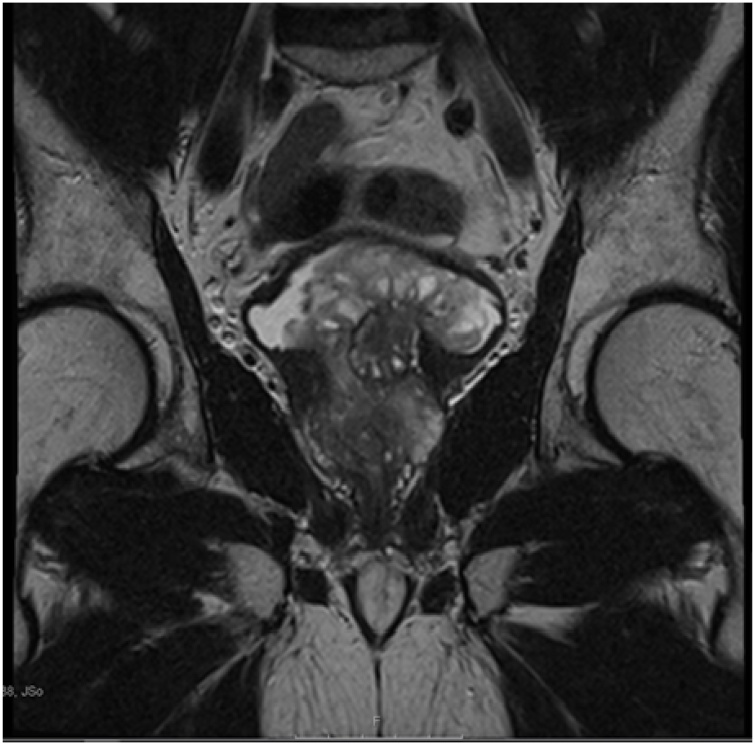
T2-weighted MRI scan (coronal view) shows the regrowth of the prostate gland into 131 ml protruding to the bladder base after only one year of TURP.

Due to the high PSA density, a trans-perineal prostate biopsy was undertaken. This was performed from 6 sectors of the peripheral zone showing 24 cores with no evidence of malignancy. The patient underwent a Trans-urethral resection of the prostate (TURP) for his obstructive symptoms in August 2016. Intraoperatively, enlarged adenoma causing bladder outlet obstruction was seen by the cystoscope and 11.85 g of prostate tissue was resected. The histopathology report confirmed the previous biopsy results of no malignancy but added that the prostate had adenomatous hyperplasia. Initially, the patient’s symptoms improved, and his PSA fell from 25 ng/ml to 3 ng/ml. A year later, the patient presented with haematuria and recurrence of his voiding LUTS. Even though his PSA was still 3 ng/ml, his prostate was much more enlarged on Ultrasound scan with a size of 131 ml. An MP-MRI scan was repeated which demonstrated a huge prostate (>100 ml) bulging into the bladder base, extending from the central gland, with a papillary middle lobe area measuring up to 6 × 5.4 x 3.9 cm intravesically, the capsule was intact, the seminal vesicles were atrophic with the same PIRAD score of 2 {Fig. 3, Fig. 4}.

The patient’s case was discussed at a multi-disciplinary prostate meeting and booked for a Holmium Laser Enucleation procedure (HoLEP) of the prostate to relieve the bladder outflow obstruction and attempt to resect all of the recurrent adenoma. The endoscopic view showed a multilobed mass which stemmed from the verumontanum and extended to the bladder neck and inside the bladder. During the procedure 64 g of tissue was removed and the histopathology report stated the specimen showed hypercellularity of the stroma, with some areas with a myxoid background, and some areas showing a spindle cell morphology. There is however, no atypia, no mitoses and no necrosis. There is also marked epithelial hyperplasia, without atypia, which are the features of Stromal Tumour of Uncertain Malignant Potential (STUMP) with no evidence of any malignancy.

**Fig. 5:**
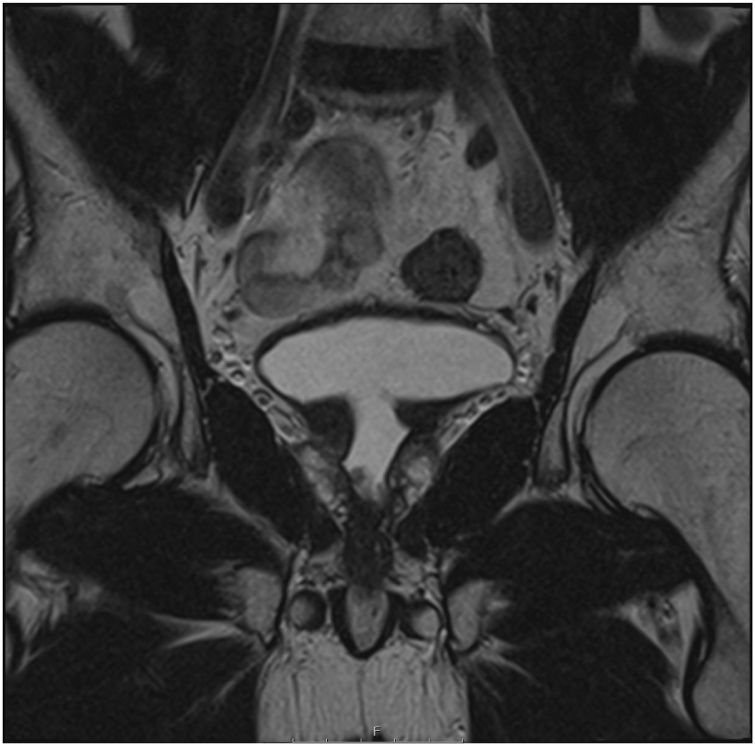
T2-weighted MRI scan (coronal view) shows no recurrence of the prostate after 1 year of the HoLEP.

The patient was followed up after 6 and 11 months of the HoLEP; the patient was not complaining of any symptoms; the PSA levels were undetectable and the MP-MRI showed no sign of recurrence or regrowth of the STUMP {Fig. 5}.

## 3 Discussion

STUMP is a rare prostate specific stromal tumour. It was initially described in 1998 by Gaudin et al. and categorized on the basis of the histopathological features of the tumour [3]. The largest case series written on STUMP of the prostate was by Herawi and Epstein in 2006, which provided a clinicopathological follow up for 50 cases of Prostate specific stromal tumours, of which 36 were STUMP. It showed that the mean age of patients with STUMP was 58 (range: 27–83) [4], which was similar to the age in this case; 57 years old. The clinical symptoms at presentation caused by STUMP vary between voiding/obstructive LUTS, haematuria, haematospermia, urinary retention, rectal dysfunction and abnormal DRE [[3], [4], [5]], which relates to our case where the patient presented with urinary obstructive symptoms of hesitancy, poor flow, incomplete bladder emptying, benign-feeling enlarged prostate on DRE and an episode of urinary retention.

Regarding the PSA levels, in this case, the patient presented with high levels of 14 ng/ml that kept rising until it reached 25 ng/ml. Other cases reported similar high PSA levels [3,4,6], and in one case it reached 500 ng/ml [7]. On the other hand, some case reports showed normal PSA levels [8]. What makes this case unique, is the unusual aggressive rapid recurrence of the tumour after TURP, where it regrew from 41 ml to 131 ml in less than a year with a huge invasion to the bladder base as seen in {Fig. 3, Fig. 4} despite removing 11 g of the tumour.

According to previous reported cases Stump showed an unpredictable clinical behaviour. Recurrence was reported in 46% by Gaduin et al. [3] and in 50% of cases by G.Bostwick et al in their case series. In the latter, only 5 cases out 23 were reported to have fast recurrence in less than a year, but there was no measured size for this recurrence or imaging available [4].

In terms of management, this is the first time HoLEP was used to treat the tumour, in an attempt to enucleate the entire origin of the STUMP growth by clearing the whole transition zone which showed good response as no recurrence or regrowth were detected. Previously, standard TURP, radical and simple prostatectomy have been used to manage these tumours [1,3,4,9].

## 4 Conclusion

STUMP of the prostate is an exceptionally rare tumour, the management of which is not clearly understood in terms of clinical presentation, response to treatment and prognosis. It should be suspected in cases of rapid recurrence after bladder outflow surgery. Close follow up should always be considered and HoLEP may be used to remove the origin of the STUMP, while other radical treatment options including resection of the prostate may be needed.

## Sources of funding

None.

## Ethical approval

No ethical approval is needed for this case report.

## Consent

Written informed consent was obtained from the patient for publication of this case report and accompanying images. A copy of the written consent is available for review by the Editor-in-Chief of this journal on request.

## Author contribution

TAREQ ALTELL: Data collection, Writing the paper.

LORENZO MARCONI: Assistant surgeon in HoLEP operation.

PAUL CATHCART: Consultant urological surgeon who did the TURP operation.

BENJAMIN CHALLACOMBE: Consultant urological surgeon who did the HoLEP, he also edited the paper.

## Registration of research studies

Not relevant for this study.

## Guarantor

MR. BENJAMIN CHALLACOMBE.

## Provenance and peer review

Not commissioned, externally peer-reviewed.

None.

## Declaration of Competing Interest

None.
